# The lower expression of circulating miR‐210 and elevated serum levels of HIF‐1α in *ischemic stroke*; Possible markers for diagnosis and disease prediction

**DOI:** 10.1002/jcla.24073

**Published:** 2021-10-28

**Authors:** Mina Rahmati, Gordon A. Ferns, Naser Mobarra

**Affiliations:** ^1^ Department of Biochemistry Metabolic Disorders Research Center Faculty of Medicine Golestan University of Medical Sciences Gorgan Iran; ^2^ Brighton and Sussex Medical School Division of Medical Education Brighton UK; ^3^ Department of Clinical Biochemistry School of Medicine Mashhad University of Medical Sciences Mashhad Iran

**Keywords:** biomarker, diagnosis, HIF‐1α, Ischemic stroke, microRNA‐210, prognosis

## Abstract

**Background:**

*Stroke*, either due to *ischemia* or *hemorrhage*, causes acute neurological damages to the brain. There is shortage of reliable biomarkers for *ischemic stroke* (IS), and we therefore investigated the serum concentrations of microRNA‐210 (miR‐210) and hypoxia inducible factor‐1α (HIF‐1α), as possible diagnostic and/or prognostic markers for IS.

**Methods:**

Serum samples were acquired from 52 IS patients and their healthy counterparts at five time points: upon admission, 24 and 48 h after admission, upon discharge and 3 months later. Serum levels of miR‐210 and HIF‐1α were respectively analyzed using real time RT‐PCR and ELISA. Diagnostic and prognostic accuracy tests were performed to assess the value of suggested biomarkers.

**Results:**

IS patients demonstrated higher levels of serum HIF‐1α and lower miR‐210 in comparison to the healthy subjects. MiR‐210 was suggested to be a weak diagnostic biomarker at the time of admission (AUC = 0.61; *p* = 0.05), while HIF‐1α was an acceptable diagnostic marker for IS (AUC = 0.73; *p* < 0.0001). The higher expression of miR‐210 and lower levels of HIF‐1α were associated with better survivals in IS patients.

**Conclusions:**

Serum miR‐210 is a weak diagnostic marker of IS. Serum HIF‐1α is a better biomarker in diagnosing IS patients but further work in larger groups, including those with hemorrhagic stroke is necessary to confirm its diagnostic utility. Similarly, the prognostic potentiality of miR‐210 and HIF‐1α was acceptable but needs bigger sample size and longer follow‐up to be statistically confirmed.

## Graphical abstract

Here, we investigated the serum concentrations of miR‐210 and HIF‐1α, as possible diagnostic and/or prognostic markers for *ischemic stroke* (IS). IS patients demonstrated higher levels of serum HIF‐1α and lower miR‐210 in comparison to the healthy subjects. MiR‐210 was suggested to be a weak diagnostic biomarker at the time of admission (AUC = 0.61; *p* = 0.05), while HIF‐1α was an acceptable diagnostic marker for IS (AUC = 0.73; *p* < 0.0001). The higher expression of miR‐210 and lower levels of HIF‐1α were associated with better survivals in IS patients.

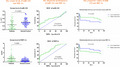

## 1 INTRODUCTION

*Stroke* is one of the principal causes of adult chronic disability and is associated with high morbidity and mortality.^1^ Stroke is divided into different pathological types; ischemic and hemorrhagic.^2^, ^3^ *Ischemic stroke* (IS) which is the most common (88% of all stroke cases), occurs when there is an occlusion in one of the cerebral arteries,^4^ leading to ischemia and loss of brain function. Accordingly, rapid treatment, and improved outcomes, depend on a reliable diagnosis. The current definitive diagnosis for IS relies on invasive methods (analysis of cerebrospinal fluid), or methods that require expensive technology such as positron emission tomography (PET).^5^ There remains a lack of established, sensitive, and specific non‐invasive methods such as serum biomarkers for the diagnosis and predicting the outcome of IS.

Noncoding RNAs (ncRNAs) play significant roles in controlling gene expression and physiologic cellular functions.^6^ These ncRNAs are highly expressed in mammalian brains and their alterations appear to have a remarkable role in brain ischemia and post‐stroke improvement.^7^ Recent reports have shown that a small, endogenous class of ncRNAs known as microRNA (17–25 nucleotides; miRNA)^8^ can modulate the transcription or translation of genes and thus regulate the expression of mRNAs and proteins.^9^, ^10^ MiRNAs are extensively stable in plasma, serum, urine, and CSF and could be introduced as diagnostic, prognostic, or remedial biomarkers for many diseases.^4^, ^11^

One of the most important groups of miRNAs, upregulated by hypoxia, is called ‘*Hypoxamirs*’.^12^ Hypoxia, the situation of inadequate oxygen nourishment to tissues, results from a reduction in oxygen availability, insufficient oxygen transport, or disability of tissues to utilize oxygen. Hypoxia is crucially involved in both acute and chronic ischemia. MiRNA‐210 (miR‐210) (MI0000286) is one the most predominant hypoxiamirs,^13^ activated by hypoxia inducible factor‐1 (HIF‐1).^14^ It has been proposed that miR‐210 could be a novel circulating biomarker for the diagnosis and prognosis of IS.^15^ Studies have also indicated that miR‐210 is a crucial determinant in adjusting several hypoxia‐associated pathways in physiological and pathological conditions.^16^ MiR‐210 can prevent apoptosis by targeting multiple transcripts involved in many sides of cellular responses to hypoxia, protect stem‐cell survivance, terminate mitochondrial metabolism, promote glycolysis, and induce angiogenesis.^17^

Enhanced HIF‐1 expression, a major characteristic of response to hypoxia, is a member of hypoxia inducible factors (HIFs). In hypoxia, the alpha subunit of HIF‐1 (HIF‐1α) is stabilized and upregulated.^18^ Targetscan information has predicted that miR‐210 could be the target of HIF‐1α (http://www.targetscan.org/vert_72/) and its expression could be regulated by hypoxia inducible transcription factors upon exposure to low oxygen.^14^, ^19^ The aim of this study was to examine the alterations of miR‐210 and HIF‐1α in patients with IS, assessing their diagnostic and prognostic values and monitoring their expressional changes in a 3 months follow‐up.

## 2 MATERIALS AND METHODS

### 2.1 Study subjects and Blood sample collection

We enrolled fifty‐two IS patients, consecutively, after following the confirmation of the diagnosis by the neurologist at Sayyad Shirazi Hospital, Gorgan, Iran, since May 2019 until November 2019. Routine physical and laboratory examinations were performed including blood glucose (BG), homeostasis function, blood pressure (BP), PT, PTT, WBC, RBC, MCV, MCH, Hb, and PLT. The verification of ischemic stroke was made by an experienced neurologist on the basis of patient's history, neurological deficit according to World Health Organization (WHO) criteria,^20^ magnetic resonance imaging (MRI), and diffusion n feature and magnetic resonance angiography (MRA) results. The study was approved by the Ethics Committee at Golestan University of Medical Sciences (GoUMS) (code of ethics: IR.GOUMS.REC.1395.23). Patients with a history of cancer, hematologic disorders, renal or liver failure, or recurrent stroke were not included in the study. Serum samples were also obtained from 52 age‐ and sex‐matched volunteers. The latter were healthy controls, who attended the Deziani Specialized and Sub‐Specialized Clinic in Gorgan, Iran for a routine health check‐up. Individuals with cardiovascular disorders, history of stroke, or kidney or liver failure, and diabetes were excluded. Written informed consent was obtained for all participants, in accordance with the declaration of Helsinki [20]. The demographic and laboratory data of all participants are reported in our previous research work [21]. The outcome of the disease was followed for 3 months after initial IS attack by the modified Rankin Scale (mRS). The mRS is a measure that evaluates disability in patients with stroke to check their recovery or continued disability over time. A score of 0 indicates no disorder, while 5 demonstrates a condition that indicates that care is constantly required, and a patient with 6 score is deceased.^21^ The mRS scores were considered as favorable (0–2) and unfavorable (3–6) outcomes. National Institute of Health stroke scale (NIHSS) scores were also used for the quantification of neurological deficit. Blood samples were collected in a five‐step procedure; at the time of admission, and then after 24 and 48 h after the stroke event, at the time of discharge, and 3 months later. Samples were centrifuged to remove blood cells, and the serum was immediately divided into two aliquots. One aliquot was immediately processed for the total RNA extraction and the other one was stored at −80°C until use.

### 2.2 RNA extraction and Real Time PCR

We isolated total RNA using TRIzol LS reagent (Invitrogen) according to the manufacturer's instructions. The spectrophotometer (Picodrop, UK) was used to quantify the integrity and concentration of total RNA. Reverse transcription using a special miR‐210 and endogenous control U6 stem‐loop primer was accomplished according to the protocols of the thermo scientific kit (K1622). Briefly, one µg of total RNA was reverse transcribed to cDNA with 2 µl of 10 mM dNTPs (with dTTP), 200 U reverse transcriptase, and 20 U RNase inhibitor in the presence of specific miRNA‐210 or U6 stem‐loop reverse transcriptase primers in a 4 µl of 5x reaction buffer and DEPC water, following the thermal cycling program of 25°C for 5 min, 45°C for 60 min, and 70°C for 5 min. The synthesized cDNA was stored at −20°C until use.

Real time quantitative PCR was performed using a fast real time PCR system (lightcycler96) using a Real Q Plus Master Mix Green (Ampliqon). Overall reaction volume was 20 µl including the components as listed: miRNA‐210 or U6 RT reaction product (3 µl), specific forward primers [miRNA‐210 (5'‐GTGTGCGTGTGACAGTGG‐3') and U6 (5'‐AAGGATGACACGCAAATTC‐3')] (1 µl), reverse primer (5'‐GAGCAGGGTCCGAGGT‐3') (1 µl), Real Q Plus Master Mix Green (10 µl), and DEPC water (5 µl). All specific primers, including miR‐21 and U6, were designed and made by Bon Yakhteh biological company (Bon Yakhteh). A 96‐well plate was then run using the following protocol; 95°C for 15 min, followed by 40 cycles of 95°C for 30 s, 60°C for 30 s, and then 72°C for 30 s. Moreover, we routinely applied three controls in all experiments; a no template control (NTC), a no reverse transcriptase control (NRT), and a no amplification control (NAC). Finally, the relative miR‐210 level was normalized to the endogenous control U6 expression for each sample in triplicates and was calculated by the 2^−Δct^ method, as previously described by Livak et al.^22^, ^23^

### 2.3 HIF‐1α ELISA assay

Serum HIF‐1α was measured using a commercial ELISA kit (Elabscience) following the manufacturer's protocol. The limit of detection (LOD) for the utilized ELISA kit was 62.50–4000 pg/ml, while the sensitivity was 37.50 pg/ml, and both intra‐ and inter‐CV (coefficient of HIF‐1α) were less the 10%, as disclosed. All samples were analyzed in duplicates and the results were reported as nanograms (ng) of HIF‐1α per mL. Linear regression was used to quantify the absorbance of 450 nm against the reference absorbance of 630 nm using a Chromate 4300 microplate reader (Biotek).

### 2.4 Statistical analyses

The study sample size was calculated based on research by C.S. Gan et al.^24^ The power of the test was also calculated to be 90%. The Kolmogorov‐Smirnov test for goodness of fit was utilized to test the normality in this study. In order to evaluate the diagnostic utility of each variable, ROC curve analyses were conducted. Pearson and Spearman correlation studies were applied to correlate quantitative variables. Logistic regression was performed to demonstrate the combined ROC curve analysis and predict the performance of miR‐210 + HIF‐1α. Linear regression was used to model the relationships between a scalar response (dependent variable) and one or more explanatory variables (independent variables). Performing the comparisons between the means of more than two groups, we used Two‐way ANOVA or Kruskal–Wallis and relevant *post hoc* tests. Kaplen–Meier survival test was used to evaluate the prognostic value for a certain amount of time after stroke after following the outcome of the disease (death or survival). The level of significant *p*‐values was considered to be 0.05. GraphPad (Prism 6 for Windows, Version 6.07) and SPSS software (Windows version 22.0), were used to perform statistical measures.

## 3 RESULTS

### 3.1 The serum concentration of miR‐210 and HIF‐1α

**FIGURE 1:**
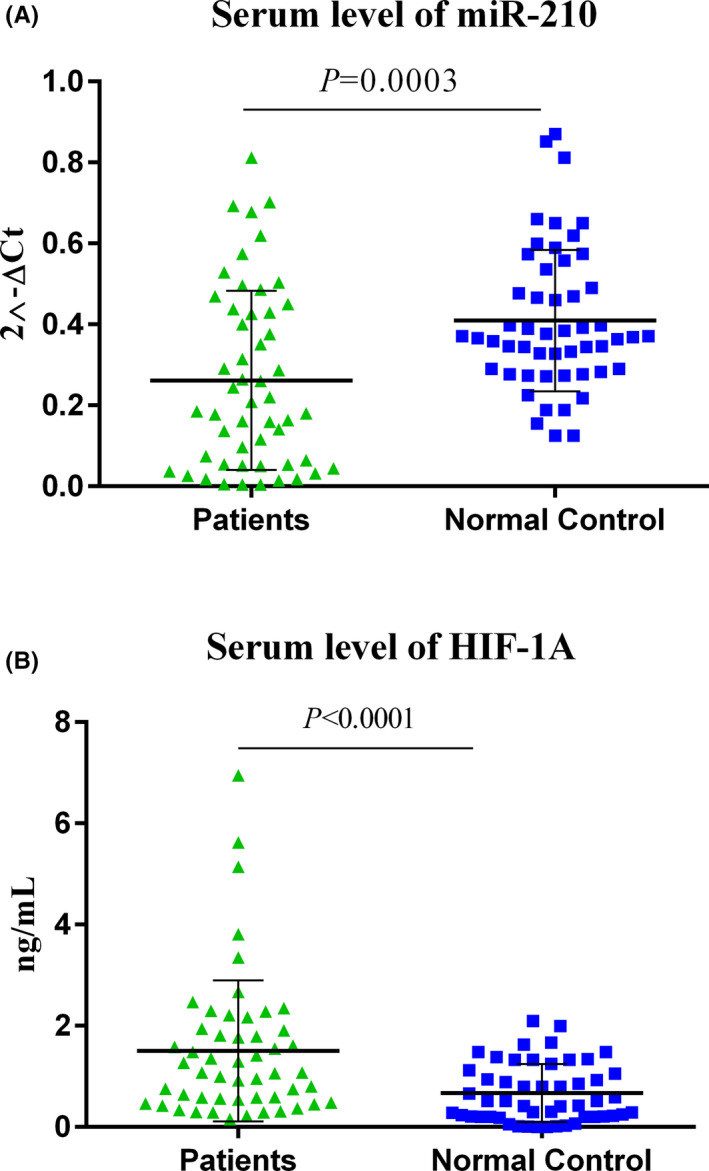
The serum steady‐state concentration of miR‐210 and HIF‐1α levels; miR‐210 was down‐regulated in IS patients (A), while HIF‐1α was expressed in higher quantities (B). The calculations of the expression levels for miR‐210 were conducted by 2^−ΔCt^ method. To compare the means between the two groups, students’ T‐test was used (Patients: 52, Healthy subjects: 52). Statistics on each scattered plot demonstrates Mean ± SD. The level of significant *p*‐values was 0.05

**FIGURE 2:**
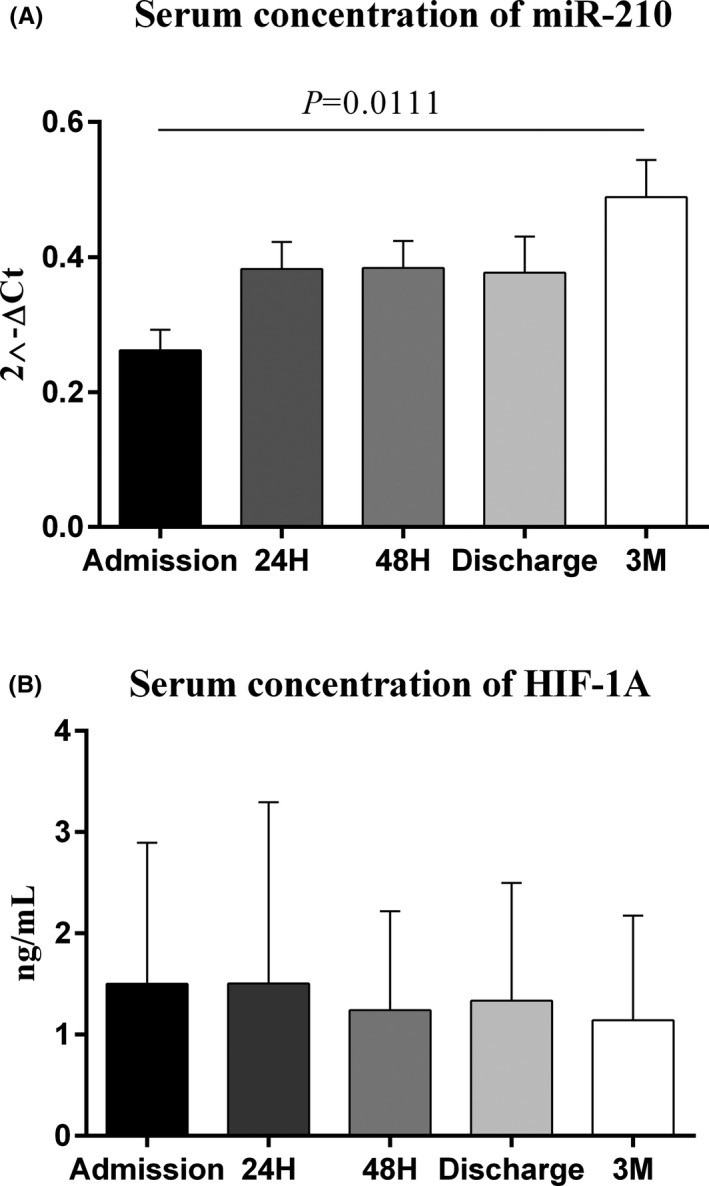
The serum steady‐state concentration of miR‐210 and HIF‐1α in 5 different time points; miR‐210 serum levels significantly altered between time of admission and 3 months after stroke (0.0111) (A), while HIF‐1α serum levels did not change significantly in all 5 time points among IS patients (B). The calculations of the expression levels for miR‐210 were conducted by 2^−ΔCt^ method. One‐Way ANOVA and Tukey's *post*‐*test* or Kruskal–Wallis and Dunn‐Bonferroni *post*‐*test* were used to evaluate the differences between the means of various groups. (Patients: 52, Healthy subjects: 52). Bar charts show Mean ± SD for each value. Level of significant *p*‐values was 0.05

Serum miR‐210 in the IS patients was lower than the normal controls, at the time of admission (*p* = 0.0003) (Figure 1A). Serum HIF‐1α was higher at the time of admission in patients compared with the normal controls (*p* < 0.0001) (Figure 1B). Serum levels of miR‐210 were significantly different between the time of admission and 3 months after the stroke incidence (*p* = 0.0111) (Figure 2A), but serum HIF‐1α did not markedly change at different time points (Figure 2B).

### 3.2 Diagnostic performance of miR‐210 and HIF‐1α

**FIGURE 3:**
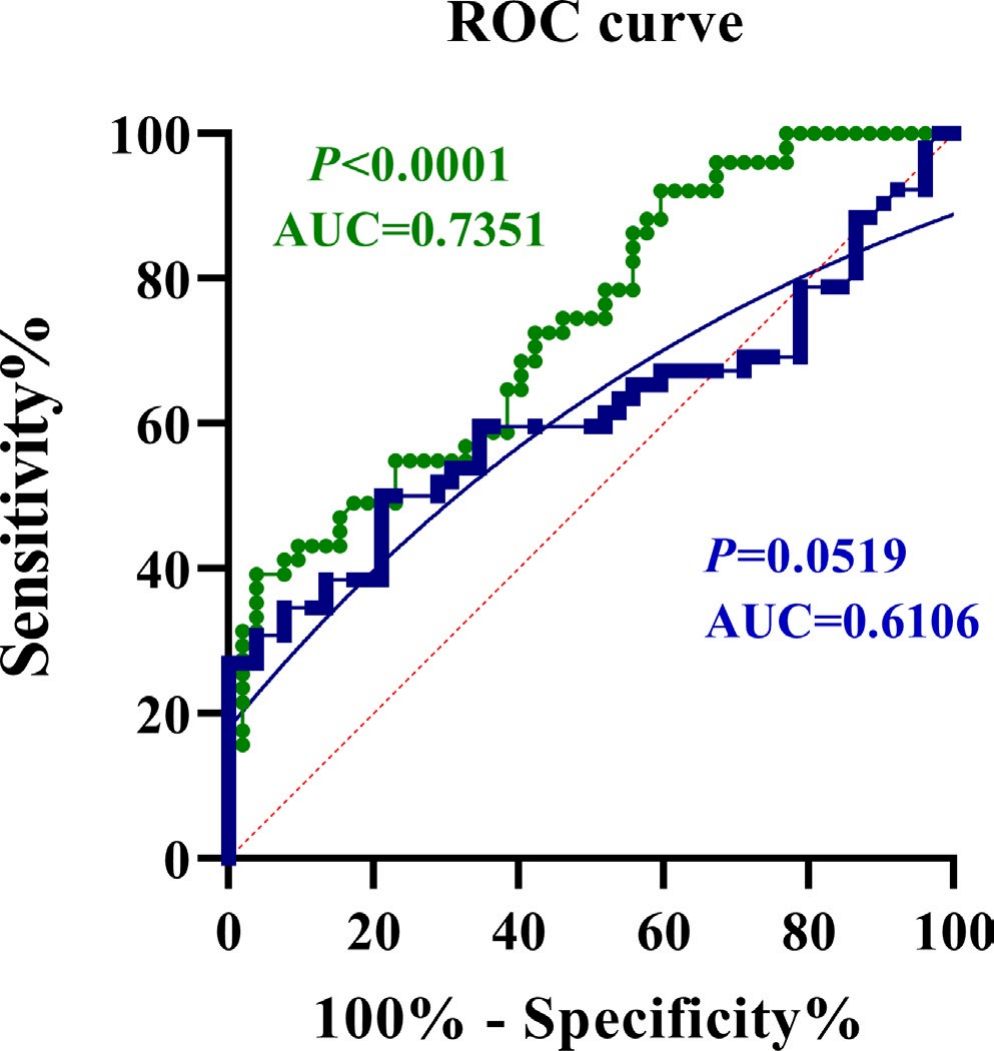
ROC curve analyses; area under the curve (AUC) for miR‐210 (BLUE symbols and connecting lines) was 0.6106 (*p* = 0.0519). Setting the cut‐off value at 0.1290 gave a sensitivity of 59.62%, specificity of 65.38% and likelihood ratio (LR) of 1.722 (A). AUC for HIF‐1α (GREEN symbols and connecting lines) was 0.7351 (*p* < 0.0001). The optimum cut‐off value at the level of 0.7995 gave a sensitivity of 64.71%, specificity of 61.54% and likelihood ratio (LR) of 1.682 (B). Logistic regression was also performed to demonstrate the combined ROC curve analysis and predict the performance of miR‐210 + HIF‐1α to diagnose IS, which is shown as a sigmoid GREY curve in the graph. All values were the expression levels at the time of zero (initial referring to the hospital)

The area under the curve (AUC) for serum miR‐210 was 0.61 (95% confidence interval (CI): 0.49–0.72; *p* = 0.05) with 59.62% Sensitivity (95% CI: 45.10%–72.99%) and 65.38% Specificity (95%CI: 50.91%–78.03%). The cut‐off point was set at the fold change level of 0.26 and likelihood ratio (LR) of 1.72 (Figure 3: BLUE symbols and connecting lines). The AUC for serum HIF‐1α was 0.73 (95% CI: 0.64–0.82; *p* < 0.0001), with a sensitivity of 64.71% (95% CI: 50.07%–77.57%) and specificity of 61.54% (95% CI: 47.02%–74.70%). The cut‐off value was set at the optimum level of 0.79 ng/ml and likelihood ratio (LR) of 1.68 (Figure 3: GREEN symbols and connecting lines). Logistic regression was also performed to demonstrate the combined ROC curve analysis and predict the performance of miR‐210 + HIF‐1α to diagnose IS, which is shown as a sigmoid GREY curve in the graph.

### 3.3 The prognostic value of miR‐210 and HIF‐1α

**FIGURE 4:**
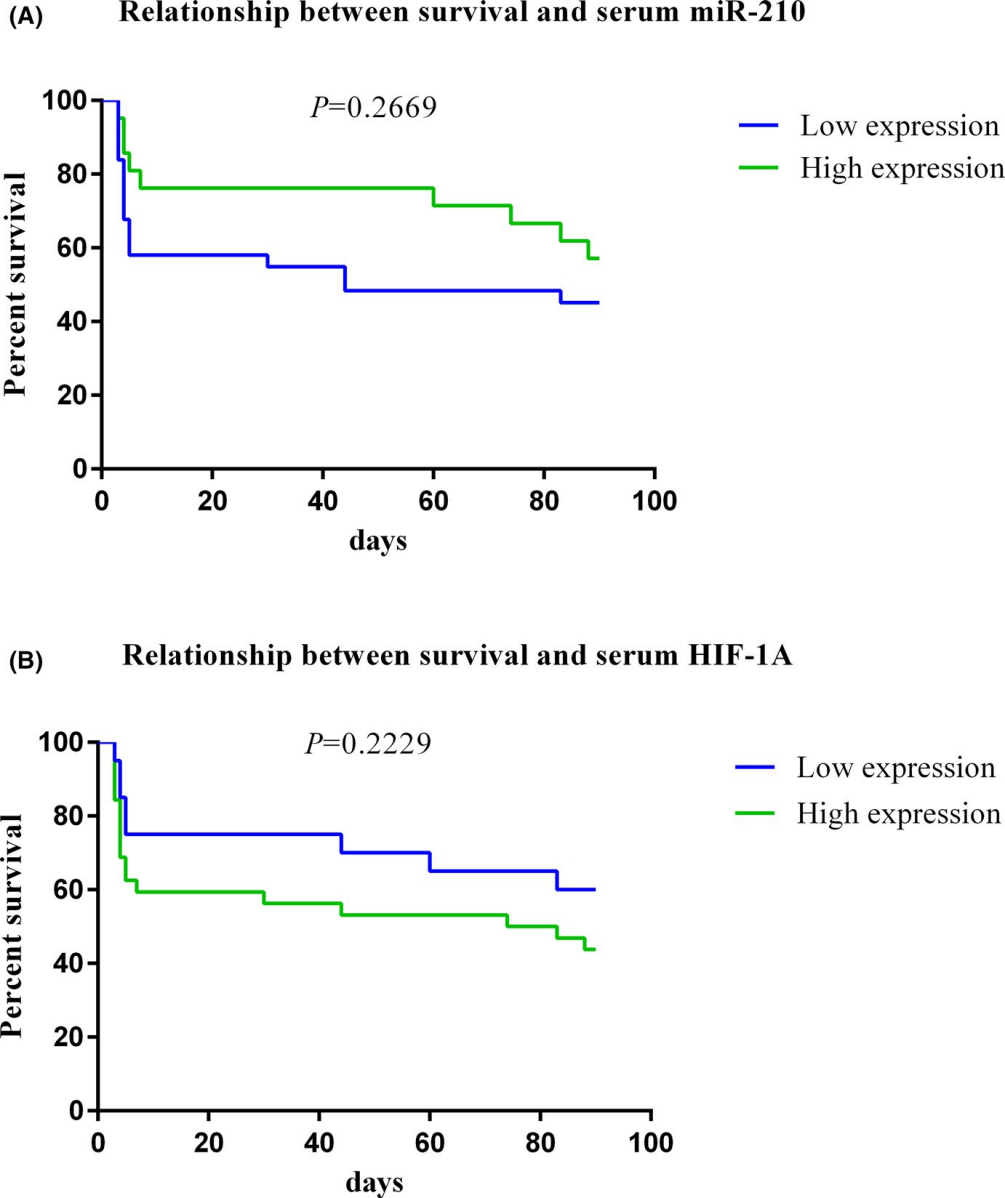
Survival analyses; IS patients were subdivided into two categories of high and low expression on the basis of the optimum cut‐off points of suggested biomarkers. Log‐rank test results showed that higher expression of miR‐210 was able to predict the outcome (survival) of the disease after 90 days of follow‐up (A) also, lower expression of HIF‐1α was capable of predicting the outcome of IS (B)

According to the outcome of the disease after 3 months of follow‐up, patients were divided into 2 groups; those who died (mean age = 73.5 years) and those who survived (mean age = 62.8). Based on the serum concentrations of both potential biomarkers, subjects were divided into categories of high and low levels according to the optimum cut‐off points derived from ROC curve analyses. Log‐rank test results revealed that higher expression of miR‐210 and lower levels of HIF‐1α were associated with better survival in IS patients (Figure 4A,B).

### 3.4 Correlation analyses

**FIGURE 5:**
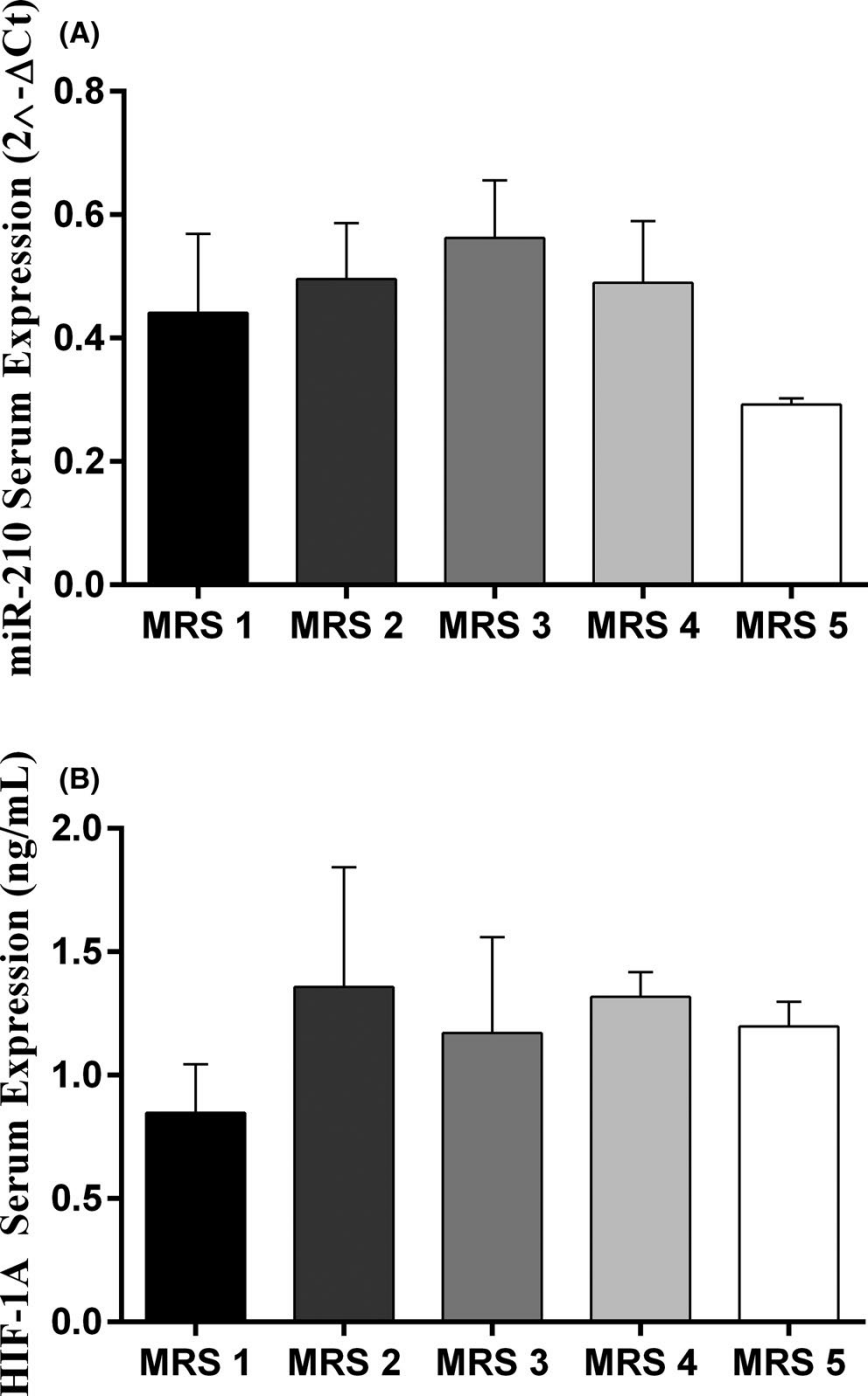
Alterations of HIF‐1α and miR‐210 after 3 months of follow‐up in different categories of mRS; miR‐210 was down‐regulated in IS patients of the MRS 5 category (A), while no remarkable change was observed in the expression of HIF‐1α between 5 groups of MRS (B). IS patients falling in the 6^th^ category of mRS had passed away and no record of HIF‐1α or miR‐602 was available. One‐Way ANOVA and Tukey's *post*‐*test* or Kruskal–Wallis and Dunn‐Bonferroni *post*‐*test* were used to evaluate the differences between the means of various groups. Bar charts show Mean ± SD for each value. Level of significant *p*‐values was 0.05

According to the mRS scores, patients were divided into 5 groups. We found that serum miR‐210 (Figure 5A) and HIF‐1α (Figure 5B) levels were not different between patient groups with different mRS scores. Moreover, a weak negative correlation was found between miR‐210 and HIF‐1α at the time of admission (rs = −0.23; *p* = 0.01).

## 4 DISCUSSION

*Ischemic stroke* is caused by the occlusion of one or more cerebral arteries, leading to hypoxia,^25^, ^26^ and resulting in a diminution of oxygen supply to the brain.^7^ Circulating microRNAs and diseases‐associated blood proteins may be helpful in discriminating patients with and without disease, and predicting outcomes.^27^ MiR‐210 is a *Hypoxamir* that is activated by hypoxia inducible factor‐alpha (HIF‐1α).^15^, ^25^ Given the role of hypoxia in stroke pathogenesis, the investigation of serum miR‐210 and HIF‐1α as biomarkers were addressed in our present study.

MiR‐210 was significantly lower in stroke patients compared to the control group and we also found that there was a significant difference between the mean of miR‐210 at the time of admission and 3 months after stroke. Several previous studies expressed that the lower expression of miR‐210 could increase PGE2 expression, in association with HIF‐1α higher levels.^28^ The lower expression of miR‐210 may be associated with the deactivation of PI3K/Akt pathway which mediates neuroprotective effects and the arrested signaling could promote unrestricted stroke.^29^ Moreover, miR‐210 downregulation is associated with the elevated expression of p53 tumor suppressor gene within DNA repair process, which is involved in the modification of stroke severity.^30^

Serum HIF‐1α levels were higher in stroke patients than controls. However, HIF‐1α protein expression did not change significantly at different time points. The serum levels of HIF‐1α, as the major modulator of pathophysiologic responses to hypoxia, are mainly controlled by being produced and degeneration in a cycle. The higher levels of HIF‐1α in response to hypoxia‐associated processes are controlled by an oxygen‐related pathway known as Hippel Lindau protein (pVHL).^31^ This pathway is probably deactivated during the first stages of ischemic stroke, in which the oxygen levels decline to a critical level.^32^ However, HIF‐1α is believed to be an early acute phase marker of IS^33^and by increasing the oxygen level, the pVHL pathway activates and consequently, the HIF‐1α serum levels fall into a consistent level.^34^ Moreover, several studies have reported that healthy individuals may express ample levels of HIF‐1α, in the absence of specific clinical diseases.^35^ This may partially explain the lack of significant difference between HIF‐1α levels at different time points, while the small sample size in our study and regular methodological errors of ELISA assay still remain major limitations that should be considered.

According to the ROC curve analyses and calculated AUC, miR‐210 could be introduced as diagnostic marker (however, with poor performance), while serum HIF‐1α might be a helpful marker with acceptable sensitivity and specificity. Although the results of Kaplan–Meier analyses were not significant, the higher expression of miR‐210 and lower levels of HIF‐1α were associated with better survival in IS patients. Moreover, serum miR‐210 and HIF‐1α had no significant relationship with NIHSS, nor with mRS at 3 months after stroke and were inversely related with each other at the time of admission. As previously reported by Zeng et al. in a 2 week follow‐up, NIHSS score was not significantly different at various measurements and no significant correlation was observed with miR‐210 expression {Zeng, 2011 #46}. Since the mean score of NIHSS in our study falls into the moderate stroke category (similar to the findings by Zeng et al.), and no significant alterations were observed in different time intervals (data not shown), the lack of correlation between miR‐210/HIF‐1α and NIHSS was predictable. Moreover, to the best of our knowledge, this was the first report investigating the NIHSS score in a 3 months follow‐up, which needs further studies to be approved. Regarding the levels of miR‐210 expression and HIF‐1α in 5 categories of mRS score, although higher mRS scores (more disabled patients) demonstrated lower levels of miR‐210 and elevated levels of HIF‐1α, the small samples size could be the reason for not achieving significant differences. Consistent with our findings, there have been several previous reports that miR‐210 is reduced in the serum of stroke patients compared to controls.^15^, ^30^, ^36^, ^37^ Other studies have also identified that miR‐210 was a powerful diagnostic marker.^15^, ^30^ Although our findings support that miR‐210 could be introduced as a diagnostic marker for IS, it is not of great value and categorized as a poor biomarker. Other investigators found that the expression of this marker in patients with mRS<2 was higher than in those with mRS>2,^36^, ^38^ while we found no significant difference between these groups.

In accordance with our findings, previous studies have shown that serum HIF‐1α concentrations were higher in stroke patients than in the control groups.^18^, ^39^, ^40^ These investigators found that concentrations of serum HIF‐1α were significantly higher in individuals with mRS>2 than in individuals with mRS≤2, but no correlation between NIHSS and HIF‐1α levels.^39^ Based on our study that HIF‐1α could be introduced as a fair diagnostic marker in addition to other diagnostic tools with less invasion. Moreover, a weak correlation was found between miR‐210 and HIF‐1α at the time of admission, which could delineate the regulatory effects of miR‐210 on the expression of HIF‐1α. The lack of association between miR‐210 and HIF‐1α after 3 months of follow‐up, could be due to the rather small sample size of our study. However, these findings are suggested to be evaluated in further investigations with bigger sample size. Since the severity of the disease was different among patients upon admission and during the study, the treatment approach was also different and it was not possible to perform subgroup analysis regarding the treatment strategies. Although our research work was associated with the limitations which are mostly affected by the small sample size, miR‐210 and HIF‐1α showed acceptable performance in predicting the fate of the disease in IS, which should be confirmed with more sophisticated further studies. It is strongly suggested to perform future research on larger sample sizes, recruiting different subgroups of ischemic stroke as control groups (at various stages, with diverse outcomes and different therapeutic approaches), and analyzing the prognosis in a longer period of time. Moreover, these suggested markers could be evaluated on other ischemic disorders to be possibly introduced as general biomarkers for ischemia.

## 5 CONCLUSION

We found that the serum levels of miR‐210 were lower, while serum HIF‐1α levels were higher in stroke patients. MiR‐210 was introduced as a poor biomarker for IS, while HIF‐1α was categorized as a fair marker in the diagnosis of IS. Moreover, both miR‐210 and HIF‐1α showed acceptable performance in predicting the fate IS patients. However, more sophisticated research works with a bigger sample size would be necessary to evaluate the diagnostic/prognostic value of these markers in distinguishing IS patients from hemorrhagic stroke. According to the significant role of Hypoxamirs including miR‐210 and targeted molecules such as HIF‐1α in ischemic disorders, our study showed that HIF‐1α and miR‐210 could be suitable diagnostic and prognostic markers for IS.

## CONFLICT OF INTERESTS

None.

## AUTHOR CONTRIBUTORS

*Mina Rahmati*: Acquisition of data, Analyses and interpretations of data, Manuscript drafting, Revision of the manuscript. *Gordon A Ferns*: Participation in data analyses and manuscript drafting. *Naser Mobarra*: Study design and concept, participation in literature bibliography, data acquisition and analyses, manuscript drafting and critical revision of the manuscript.

## ETHICAL APPROVAL

The study was approved by the Committee of Ethics, Golestan University of Medical Sciences (IR.GOUMS.REC.1395.23). All patients signed a written informed consent following the declaration of Helsinki.

## Data Availability

Data supporting the findings of this study are available from the corresponding author upon reasonable request.
